# Transfusion of stored autologous blood in patients with low-grade pseudomyxoma peritonei: A retrospective analysis of its safety and outcome

**DOI:** 10.3389/fonc.2022.1022426

**Published:** 2022-10-06

**Authors:** Xiaoyun Gao, Liduo Kou, Hang Guan, Hua Tian, Junhui Jia, Yu Bai, Wei Bai, Yanhui Di, Ruiqing Ma, Xinhua Wang

**Affiliations:** ^1^ Department of Blood Transfusion, Aerospace Center Hospital, Beijing, China; ^2^ Department of Myxoma, Aerospace Center Hospital, Beijing, China

**Keywords:** pseudomyxoma peritonei, stored autologous blood transfusion, recurrence rate, propensity score matching, coagulation function

## Abstract

**Background:**

Pseudomyxoma peritonei is a rare disease that presents as a malignant tumor on the peritoneal surface. Cytoreductive surgery combined with hyperthermic intraperitoneal chemotherapy is the standard treatment for this disease and frequently requires a red blood cell transfusion. However, due to the limited collection and supply of allogeneic blood, surgical treatment may be delayed due to inadequate preparation of allogeneic blood in the course of clinical treatment. This study aimed to evaluate the safety and efficacy of transfusion of stored autologous blood in patients with low-grade pseudomyxoma peritonei.

**Methods:**

Pseudomyxoma peritonei patients who received cytoreductive surgery combined with heat-infused peritoneal chemotherapy were divided into two groups: transfusion of allogeneic blood and transfusion of stored autologous blood. A comparison of the differences in multiple factors between the two groups was performed, including tumor recurrence, survival time, hemoglobin and hematocrit levels, coagulation function (prothrombin time, activated partial thromboplastin time, and fibrinogen), total hospital stay duration, and incidence of serious adverse events after surgery.

**Results:**

Propensity scores matching analysis yielded 34 patients with allogeneic blood transfusion and 34 patients with stored autologous blood transfusion. Comparison analysis did not show statistical differences in several factors, including age, tumor grade, tumor recurrence rate after surgery, etc., between the two groups. The cytoreductive degree was considered an independent risk factor for tumor recurrence. The pseudomyxoma peritonei patients in the autologous transfusion group had a higher 5-year survival rate and a longer survival time. Moreover, transfusion of stored autologous blood did not increase the rate of tumor recurrence, or the total hospital stay duration after surgery, the hemoglobin level and coagulation function were well stabilized within 24 h after surgery, and there was a low incidence of serious adverse events.

**Conclusion:**

The clinical application of transfusion of stored autologous blood in pseudomyxoma peritonei patients is safe and effective.

## Introduction

Pseudomyxoma peritonei (PMP) is a rare disease that presents as a malignant tumor on the peritoneal surface (1), and its primary lesion is most located in the appendix (2, 3). PMP is characterized by mucinous ascites caused by the extensive dissemination of appendiceal mucinous tumors in the abdominal and pelvic cavity, and its incidence shows an increasing trend (4, 5). Low-grade appendiceal mucinous neoplasm is the most common pathological type, accounting for 60–70% of mucinous tumors of appendiceal origin (6, 7). The current standard therapy for PMP is complete cytoreductive surgery (CRS) combined with hyperthermic intraperitoneal chemotherapy (HIPEC) (8–16). CRS includes the resections of all visible tumor tissues in the abdominopelvic cavity, and its procedures include total laparotomy, left superior laparotomy, greater omentectomy + splenectomy, right superior laparotomy, and pelvic laparotomy + rectosigmoid resection. Because CRS+HIPEC treatment is surgically complex, involving the removal of many organs, the duration of the operation is usually long and the area of resection is extensive, thus requiring large amounts of intraoperative allogeneic blood transfusions. However, surgical treatment may be delayed due to inadequate preparation of allogeneic blood, which is caused by the limited collection and supply of allogeneic blood. To avoid the shortcomings of allogeneic blood transfusion, the transfusion of stored autologous blood has been widely applied in clinical surgery and has become an important strategy for blood conservation (17–22). However, the transfusion of stored autologous blood in CRS+HIPEC has rarely been reported.

The transfusion of stored autologous blood is mainly applied to patients who need a blood transfusion for elective surgery; those with expected high intraoperative bleeding; or those with rare blood types or difficult-to-match blood. In addition, the transfusion of stored autologous blood is performed when there is difficulty in obtaining a blood supply for various reasons such as the demand for blood transfusion is too great or when patients refuse to be transfused with allogeneic blood due to religious beliefs, etc. The qualifications for receiving transfusion of stored autologous blood include that the patient is in good general condition, with preoperative hemoglobin (Hb)>110 g/L or hematocrit (HCT)>0.33; but there are no clear restrictions on the patient’s age or body mass. Currently, this strategy is an effective alternative method to allogeneic transfusion in orthopedic surgery, cardiovascular surgery, maternal delivery, and oncologic surgery. Compared to infection risks (e.g., due to blood-borne infectious diseases, such as hepatitis B, hepatitis C, and human immunodeficiency virus infection) and adverse reactions (such as hemolysis, fever, and allergy) caused by allogeneic blood as well as the increased incidence of postoperative infections, tumor recurrence, and multi-organ dysfunction syndrome related to allogeneic blood transfusion (23, 24), the transfusion of stored autologous blood has a significantly lower rate of adverse transfusion reactions (25). However, some concerns remain, such as the safety of stored autologous blood transfusion in patients with malignant tumors. Whether the application of stored autologous blood transfusion will increase the recurrence rate of malignant tumors or affect patient survival remains unknown. Previous reports have demonstrated controversial opinions. Therefore, it is necessary to clarify the safety issue of stored autologous blood transfusion, especially using stored autologous blood collected from PMP patients.

To ensure a safe and efficient intraoperative transfusion for CRS+HIPEC therapy, we investigated the safety and efficacy of the transfusion of stored autologous blood in patients with low-grade PMP in this study.

## Materials and methods

### Patients

This was a retrospective study that recruited a total of 373 PMP patients who received treatment at the Mucinous Tumor Unit of the Aerospace Center Hospital, Beijing, China, during the period from January 2015 to December 2021. All recruited patients had a clear diagnosis of PMP, based on the 2016 International Collaborative Group on Peritoneal Surface Tumors Expert Consensus (26), and underwent standardized CRS+HIPEC treatment. The primary tumors of the patients had an appendicular origin. Among the 373 recruited patients, there were 329 subjects with allogeneic blood transfusion and 44 with stored autologous blood transfusion. Those who received a combination of both transfusion modalities were excluded from this study.

### Criteria for collecting autologous blood

The enrolled PMP patients were in good general condition, aged 70 years old or younger, had Hb≥110 g/L or HCT≥0.33, and had signed the Informed Consent Form for Autologous Blood Collection. None of the enrolled patients were treated with iron supplementation or erythropoietin before blood collection. The patients with the following conditions were excluded from undergoing preoperative autologous blood collection: those with severe cardiovascular disease that could not tolerate the blood collection; those with bacteremia, sepsis, or infectious fever; those with abnormal coagulation function, platelet count<50×10^9^/L, or abnormal function; those with liver or kidney insufficiency; those with preoperative anemia; those with active epilepsy or other psychiatric diseases; and those with previous serious adverse reactions to blood donation.

The amount of autologous blood collection was determined according to the patient’s tolerance and the need for surgery. The amount of a single collection was controlled at 10–15% of the total amount of autologous blood circulation, so the amount of blood collected ranged from 200 mL to 400 mL. During the collection process of autologous blood, it was ensured that the Hb level was ≥90 g/L after the last collection, so further treatment was not necessary. The interval between two collections was at least 3 days.

### Collection and storage of autologous blood

The collection of autologous blood (with multiple batches) started two weeks before the operation. Before and after blood collection, the patient’s blood pressure, pulse, and heart rate were measured. The collection bag used for the storage of autologous blood was a Nangal single-use plastic blood bag (model NIGALE-S-200/400), which contained blood preservation solution III (CPDA-1) (28 mL or 56 mL), with a capacity of 200 or 400 mL of whole blood. The blood preservation solution III contained the following components: 3.27 g/L sodium citrate (H_2_O), 26.3 g/L sodium citrate (2 H_2_O), 31.9 g/L glucose (H_2_O), 2.22 g/L sodium dihydrogen phosphate (H_2_O), and 0.275 g/L adenine. After blood collection, the interface of the collection bag was thermally closed using a heat-sealing machine. All the collected autologous blood was examined for the ABO blood type and Rh blood type, and the results were labeled on the surface of each collection bag, which was stored in a special refrigerator for blood storage (2–6°C). The storage duration was no more than 35 days after collection. The last batch of autologous blood was collected at 72 h before the surgery.

### Allogeneic blood transfusion

The preparations of blood components for allogeneic blood transfusion were provided by the Beijing Red Cross Blood Center. The red blood cells were in a suspension of erythrocytes that were separated from whole blood by removing the plasma. In the blood preparations, the specification of blood cells was 1 or 2 U, and all transfused red blood cells were irradiated by 25–30 Gy of gamma radiation. Red cell transfusion was performed using homotypic ABO and Rh blood preparations, and a cross-matching test was done before the transfusion. The volume of the frozen plasma was 100 mL or 200 mL, which was thawed in a water bath at 37°C for 25 min before transfusion. Plasma transfusion usually followed the transfusion of the ABO and RhD blood isotype group. All of the transfused blood components were within the effective shelf life. All patients who received allogeneic blood transfusion underwent routine blood and coagulation tests within 24 h before and after blood component transfusion.

### Statistical analysis

SPSS 26.0 software was used for data analysis in this study. The analysis results of normally distributed data were expressed as the mean ± standard deviation or median (range), while the independent-sample t-test was used for comparing the differences between groups. Categorical variables were represented as a frequency or percentage. The chi-squared test was used for the comparison of differences in countable data between groups. The multiple logistic regression test was used for linear analysis of categorical data correlation. The Kaplan–Meier method was applied to calculate the overall survival, while the log-rank test was used for survival rate comparison. The Cox proportional-hazards model was used for prognostic multifactor analysis. *P*<0.05 was a statistically significant difference.

## Results

### Baseline patient characteristics

The baseline patient characteristics of the 329 allogeneic and 44 autologous blood transfusion subjects showed significant differences (*P *< 0.05) in their sex, age, cytoreductive degree (CCR), peritoneal cancer index (PCI), intravenous chemotherapy, and 24-h Hb before surgery, which may directly affect the analysis results of this study. To ensure the balance of basic clinical information between the two groups of patients, propensity score matching (PSM), a statistical matching technique, was applied in this study. Finally, 34 patients who received autologous transfusion and 34 patients who received allogeneic transfusion were identified after matching. Among these patients, there were no statistically significant differences in sex, CCR, PCI, intravenous chemotherapy, prior surgical score (PSS)(*P*=1.000), age, tumor grade, or preoperative 24-h Hb (**Table 1**).

**Table 1:** Basic characteristics of the two groups of patients with PMP before and after PSM.

Variable	Before PSM					After PSM				
	Autologous blood transfusion	Allogeneic transfusion	χ^2^		*P*	Autologous blood transfusion	Allogeneic transfusion		χ^2^	*P*
Sex										
Male	21	99		4.753	0.029^*^	22	22	0.000		1.000
Female	23	230				12	12			
Age (years old)										
≤70	44	292		4.307	0.038^*^	0	0	0.000		1.000
>71	0	37				34	34
PSS										
0–1	25	143		2.282	0.131	18	18	0.000		1.000
2–3	19	186				16	16
CCR										
0–1	32	53		67.524	0.000^*^	22	22	0.000		1.000
2–3	12	276				12	12
PCI										
≤20	32	80		41.017	0.000^*^	22	22	0.000		1.000
>21	12	249				12	12			
Pathology										
0–1	39	303		0.240	0.624	34	34	0.000		1.000
2–3	5	26				0	0
Prior chemotherapy										
Yes	4	97		7.173	0.007^*^	4	4	0.000		1.000
No	40	232				30	30
Hb (24 h before operation)										
≤90 g/L	0	79		12.005	0.001^*^	0	0	0.000		1.000
>91 g/L	44	250				34	34

PMP, pseudomyxoma peritonei; PSM, propensity score matching; CCR, cytoreductive degree; PCI, peritoneal cancer index; Hb, hemoglobin; PSS, prior surgical score; *P < 0.05.

### Postoperative tumor recurrence rate and survival analysis

**Figure 1:**
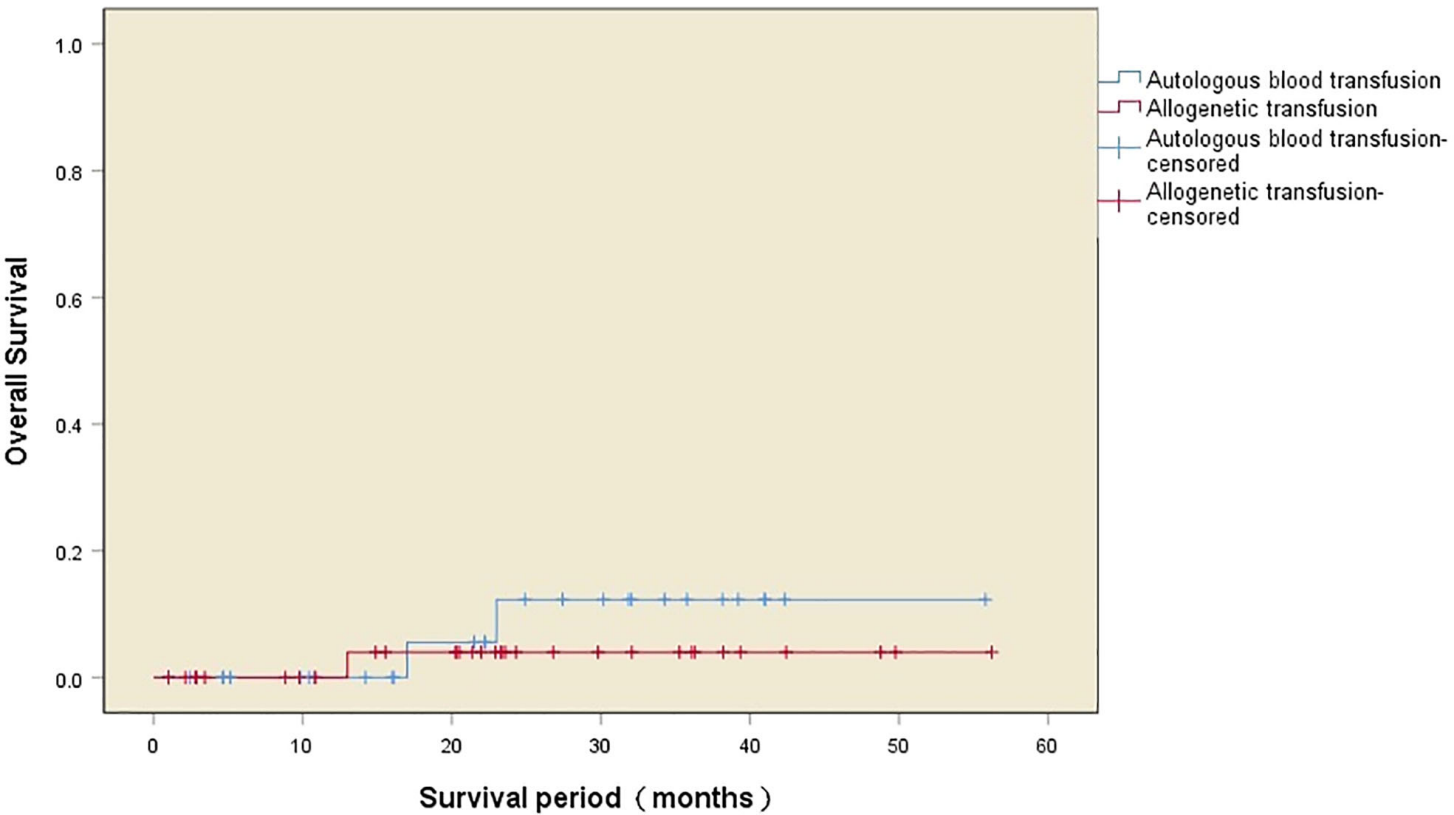
Kaplan–Meier curves of the cumulative tumor recurrence rate after the operation in the two groups.

All patients in the autologous transfusion and allogeneic transfusion groups were followed up successfully. During the period of follow-up, two patients in the autologous transfusion group and one patient in the allogeneic transfusion group had tumor recurrence. There was no significant difference in the recurrence rate between the two groups (*P*=0.457). The cumulative tumor recurrence rates of the two groups after surgery are shown in **Figure 1**. A multifactorial analysis of the variables affecting postoperative recurrence in both groups recognized CCR as an independent risk factor for tumor recurrence after surgery in patients with low-grade PMP (*P*=0.003) (**Table 2**).

**Table 2:** Multivariate correlation analysis of the factors affecting postoperative recurrence.

Variable	B	*P*	Exp (B)	95% CI of Exp (B)	
Sex	-12.813	0.956	2.726E-6	4.595E-203	1.617E+191
Type of transfusion	0.572	0.711	1.771	0.086	36.470
PSS	11.559	0.958	104663.491	1.745E-184	6.277E+193
Prior chemotherapy	11.297	0.974	80570.020	2.575E-293	2.521E+302
Blood loss	11.150	0.980	69569.550	0.000	–
Blood transfusion	0.306	0.879	1.357	0.026	70.221
CCR	5.833	0.003^*^	341.547	7.090	16452.772
PCI	-1.371	0.275	0.254	0.022	2.976

PSS, prior surgical score; CCR, cytoreductive degree; PCI, peritoneal cancer index; *P < 0.05.

**Figure 2:**
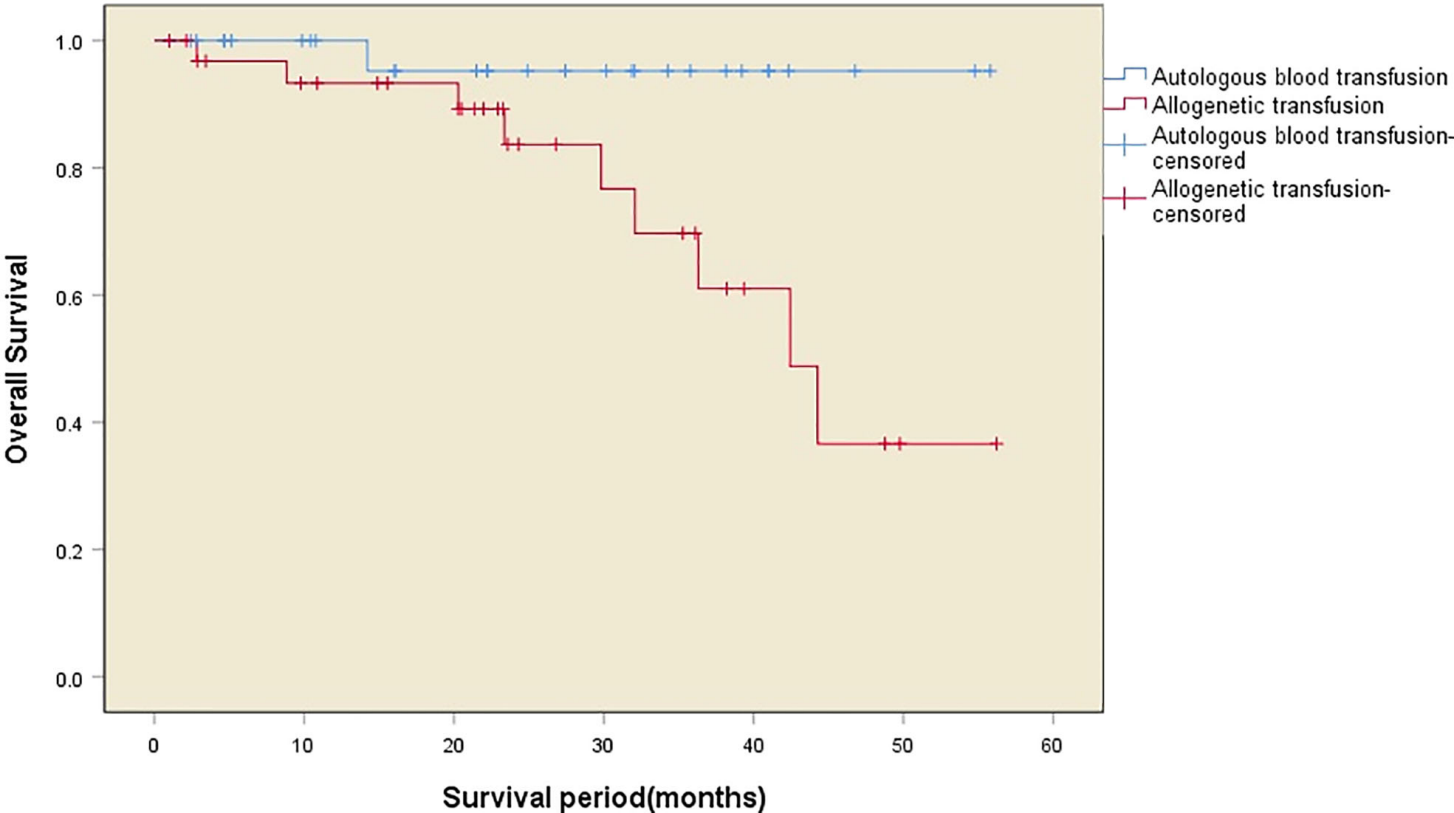
Kaplan–Meier curves showing the overall survival of the patients in the two groups.

The median survival time after surgery in the two transfusion groups was 42 (32–51) months, with one death in the autologous transfusion group and nine deaths in the allogeneic transfusion group during the follow-up period. There was a significant difference in the postoperative survival between the two groups (χ^2 =^ 5.520, *P*=0.019), with a higher 5-year survival rate (97.1% *vs*. 73.5%) and a longer survival time (54.0 ± 2.0 months *vs*. 41.2 ± 3.5 months) in the autologous transfusion group compared to the allogeneic transfusion group. The survival curves of the patients in the two groups are shown in **Figure 2**, while the independent risk factors for survival are shown in **Table 3**.

**Table 3:** Cox regression analysis of the factors affecting the postoperative survival.

Variable	B	SE	Wald	*P*	Exp (B)	95% CI of Exp (B)
Type of transfusion	-2.020	1.075	3.533	0.060	0.133	0.016–1.090
Sex	0.748	0.890	0.706	0.401	2.113	0.369–12.101
PSS	0.973	0.897	1.175	0.278	2.646	0.456–15.362
Prior chemotherapy	1.134	0.937	1.462	0.227	3.107	0.490–19.508
CCR	-0.341	1.228	0.077	0.781	0.711	0.064–7.895
PCI	1.032	1.186	0.757	0.384	2.807	0.275–28.702

PSS, prior surgical score; CI, confidence interval; CCR, cytoreductive degree; PCI, peritoneal cancer index.

### Outcomes of Hb, coagulation function, and blood transfusion

The Hb level, HCT level, and coagulation function at 24 h before and after the operation in the two groups were evaluated. Before the operation, the Hb level and coagulation functions were normal in both the autologous transfusion and allogeneic transfusion groups; however, the Hb, HCT, and prothrombin time (PT) in the autologous transfusion group were better than those in the allogeneic transfusion group, while fibrinogen (FIB) was lower in the autologous transfusion group than in the allogeneic transfusion group. There was no significant difference in the activated partial thromboplastin time (APTT) between the two groups before the operation, but there were significant differences in Hb, HCT, PT, and APTT between the two groups within 24 h after the operation (*P*=0.000). The Hb level and coagulation function of the patients with autologous blood transfusion were significantly better than those of the patients with allogeneic blood transfusion, while there was no significant difference in FIB between the two groups (**Table 4**).

**Table 4:** Coagulation function and blood transfusion of patients in the two groups before and after the operation.

Variable	Autologous blood transfusion (mean ± SD)	Allogeneic transfusion (mean ± SD)	T	*P*
Coagulation function before the operation				
FIB	2.99 ± 0.67	4.01 ± 1.03	4.832	0.000*
Hb	124.91 ± 15.10	110.26 ± 12.37	-4.375	0.000*
HCT	0.37 ± 0.04	0.34 ± 0.03	-3.134	0.003*
PT	11.47 ± 0.92	12.28 ± 1.04	3.410	0.001*
APTT	31.17 ± 3.29	32.09 ± 5.25	0.863	0.391
Coagulation function after the operation				
FIB	2.61 ± 0.82	2.58 ± 0.63	-0.195	0.086
Hb	121.50 ± 15.27	101.41 ± 13.25	-5.974	0.000*
HCT	0.37 ± 0.05	0.31 ± 0.04	-5.608	0.000*
PT	13.68 ± 1.97	16.57 ± 3.40	4.299	0.000*
APTT	30.24 ± 4.24	36.97 ± 8.94	3.970	0.000*
Blood transfusion				
Red blood cells (U)	0	3.94 ± 2.28	10.069	0.000*
Plasma (mL)	0	408.82 ± 253.90	9.389	0.000*
Autologous whole blood (mL)	535.29 ± 254.51	0	12.264	0.000*
Time from collection to transfusion of red blood cells (d)	6.53 ± 15.30	15.94 ± 6.80	3.278	0.002*

FIB, fibrinogen; Hb, hemoglobin; HCT, hematocrit; PT, prothrombin time; APTT, activated partial thromboplastin time; SD, standard deviation; *P < 0.05.

A total of 18200 mL of autologous blood was collected from 34 patients with autologous blood transfusion before the operation, and all of them were retransfused during the operation. The patients in the autologous blood transfusion group were transfused only with stored autologous whole blood, rather than allogeneic blood during the operation. The average volume of stored autologous whole blood used for transfusion was 535.29 ± 254.51 mL. In the allogeneic blood transfusion group, the average amount of allogeneic red blood cells for transfusion was 3.94 ± 2.28 U. The volume of plasma for transfusion was 408.82 ± 253.90 mL. There was a significant difference in the amount of blood transfused between the two groups (*P*<0.05). In addition, the blood collection–transfusion time periods were significantly different between the two groups (*P*=0.002), which were 6.53 ± 15.30 days in the autologous blood transfusion group and 15.94 ± 6.80 days in the allogeneic blood transfusion group (**Table 4**).

### Surgery-related indicators and serious adverse events

**Figure 3:**
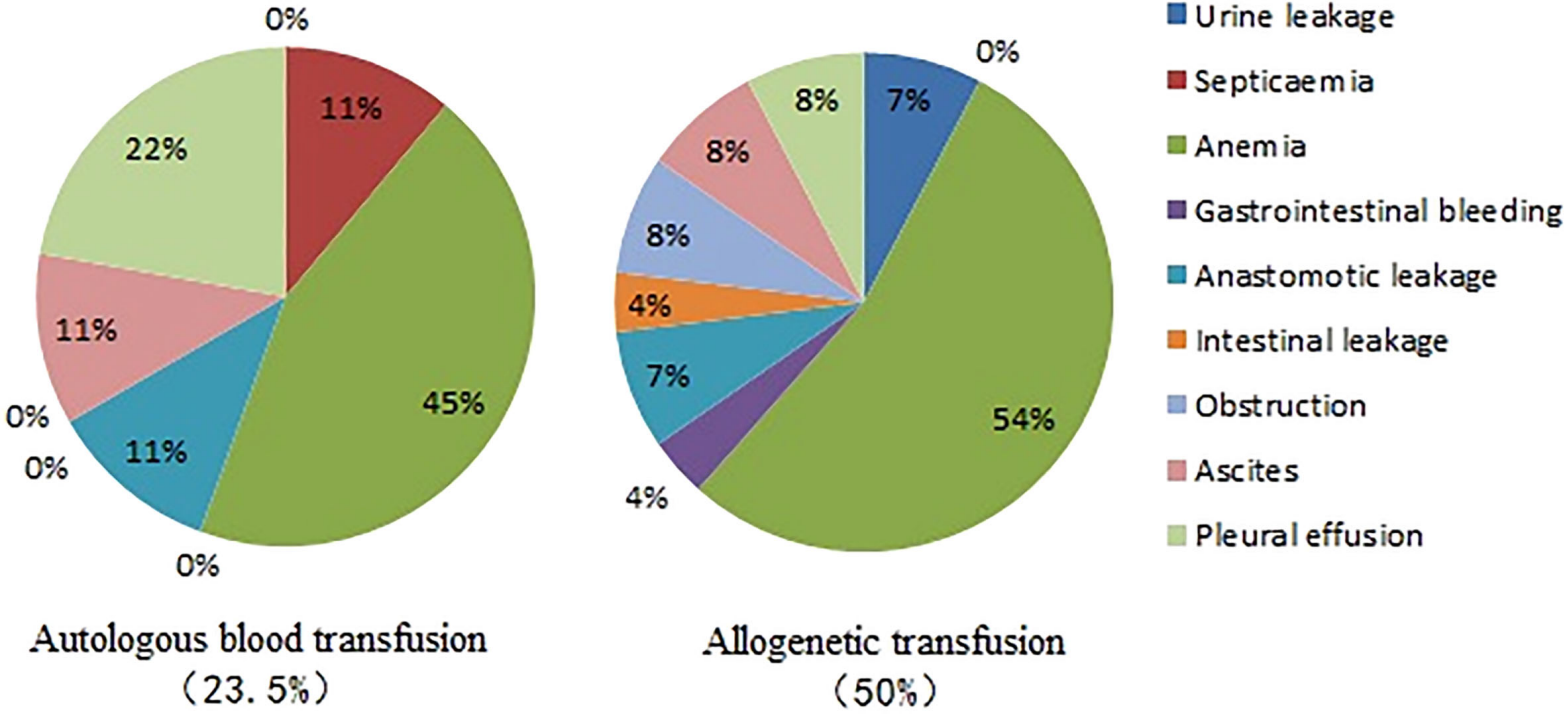
Distribution of serious adverse events in the two groups.

There were significant differences in the total hospital stay duration, operation duration, number of organ resections, and incidence of serious adverse events between the patients in the two groups (*P*<0.05). The total hospital stays and operation durations of the patients with autologous blood transfusion were shorter than those of the patients with allogeneic blood transfusion. The number of organ resections and the amount of intraoperative bleeding in the patients with allogeneic blood transfusion were more than those in the patients with autologous blood transfusion. However, there was no difference in the number of peritoneal resections between the two groups. The incidence of serious adverse events in the autologous transfusion group was lower than that in the allogeneic transfusion group (23.5% *vs*. 50%) (**Table 5**). The distributions of serious adverse events for the two groups are shown in **Figure 3**.

**Table 5:** Perioperative data of patients in the two groups.

Variable	Autologous blood transfusion	Allogeneic transfusion	t/χ^2^	*P*
Hospital stays duration (d)	24.56 ± 6.03	31.00 ± 13.72	2.506	0.015*
Operation duration (min)	449.29 ± 105.19	539.79 ± 124.13	3.143	0.002*
Peritoneum removal	4.00 ± 2.19	4.41 ± 2.52	0.719	0.475
Organ removal	3.50 ± 1.69	4.53 ± 2.23	2.143	0.036*
Blood loss (mL)	597.06 ± 364.72	1694.12 ± 1038.41	5.812	0.000*
Serious adverse events (*n*)	8 (23.5%)	17 (50%)	4.048	0.044*

*P < 0.05.

## Discussion

This study aimed to evaluate the value of preoperative autologous blood storage used for blood transfusion for PMP treatment by CRS, through a retrospective analysis of the safety and outcome of stored autologous blood transfusion. The following concerns were addressed in our study. First, whether stored autologous blood transfusion would increase the recurrence rate of malignant tumors and affect patient survival. Second, whether stored autologous blood transfusion would increase the chance of postoperative infections and adverse reactions (23, 24). Third, whether stored autologous blood transfusion would prolong the length of hospitalization, which is a risk factor for death in critically ill patients (27).

In this study, we compared the differences in tumor recurrence, postoperative survival time, occurrence of serious adverse events, and changes in blood transfusion and coagulation function between 329 allogeneic blood transfusion patients with low-grade PMP tumors and 44 autologous blood transfusion patients. In addition, the PSM method was used to ensure the balance of the two groups. Our results showed that the postoperative tumor recurrence rates of patients with low-grade PMP tumors were not significantly different between the allogeneic blood transfusion group and the stored autologous blood transfusion group. In fact, autologous blood transfusion did not increase tumor recurrence. The patients who received stored autologous blood transfusion had a higher survival rate and a longer survival time compared to those in the allogeneic blood transfusion group. The multi-factorial regression analysis indicated CCR as an independent risk factor affecting tumor recurrence after surgery in patients with low-grade PMP. Therefore, the removal of visible tumors as much as possible during surgery is key to reducing tumor recurrence after surgery. Previous studies have reported that preoperative storage of collected autologous blood may increase the probability of anemia in patients and may prolong hospitalization (28). However, our study showed that autologous blood collection completed three days before surgery did not increase anemia in patients. Moreover, both the Hb level and the coagulation function of the autologous transfusion patients were better than those of the allogeneic blood transfusion patients at 24 h before surgery; this finding may be because autologous blood collection can induce activation of the bone marrow hematopoietic system before and after the surgery, thereby facilitating the *de-novo* production of various blood components (29). It is notable that the stored autologous whole blood in this study had a short time interval from collection to transfusion, to ensure a better quality of red blood cells and coagulation factors; whereas the red blood cells and frozen plasma that were separated from the allogeneic whole blood had a long-time interval from collection to transfusion, which may have affected the blood quality (30). This is the likely reason why the stored autologous blood transfusion showed better results than allogeneic blood transfusion in the replenishment of red blood cells and Hb during blood loss as well as the correction and maintenance of coagulation function after surgery. In addition, the incidence of serious adverse reactions, such as postoperative intestinal leakage and gastrointestinal bleeding after surgery, in the autologous transfusion patients was lower than that in the allogeneic transfusion patients, probably because the immune system of the autologous transfusion patients received less stimulation compared to that of the allogeneic transfusion patients, who may have had a strong immune response to allogeneic blood components (31). In this study, the total hospital stay duration was also shorter for the autologous transfusion patients, suggesting that stored autologous blood transfusion is helpful for reducing treatment costs and enhancing the use of medical resources.

However, our study only focused on the safety and efficacy of using stored autologous blood transfusion for treating patients with low-grade PMP tumors. Further studies are needed to determine whether stored autologous blood transfusion is also suitable for treating patients with other types of PMP tumors. Meanwhile, although this study used the PSM method to ensure the balance between the two groups, a certain degree of selection bias could not be completely avoided due to the nature of a retrospective study, even if matching between study subjects was performed. To improve the quality of the study and to obtain a more reliable conclusion, a prospective study that has more comparable groups with a larger sample size should be conducted in the future.

## Conclusions

Taken together, the application of stored autologous blood transfusion for treating patients with low-grade PMP did not increase postoperative tumor recurrence; instead, it prolonged the survival time as well as improved the coagulation function of the patients before and after surgery. Moreover, the incidence of serious adverse events remained low for the autologous blood transfusion group, contributing to a shorter hospital duration as well as saving a large amount of allogeneic blood components. Therefore, stored autologous blood transfusion could be a better option for treating patients with low-grade PMP tumors.

## Data availability statement

The raw data supporting the conclusions of this article will be made available by the authors, without undue reservation.

## Author contributions

All authors listed have made a substantial, direct, and intellectual contribution to the work and approved it for publication.

## Funding

This study was supported by the Beijing Gold-Bridge Project (No. ZZ21043).

## Conflict of interest

The authors declare that the research was conducted in the absence of any commercial or financial relationships that could be construed as a potential conflict of interest.

## Publisher’s note

All claims expressed in this article are solely those of the authors and do not necessarily represent those of their affiliated organizations, or those of the publisher, the editors and the reviewers. Any product that may be evaluated in this article, or claim that may be made by its manufacturer, is not guaranteed or endorsed by the publisher.
